# A Comprehensive Review on the Efficacy and Safety of Vonoprazan in the Management of Gastric Acid-Related Diseases

**DOI:** 10.7759/cureus.64777

**Published:** 2024-07-17

**Authors:** Vishal Padwale, Vijendra Kirnake, Ravi Daswani, Akshay Kodmalwar, Anusha Gupta

**Affiliations:** 1 Gastroenterology, Jawaharlal Nehru Medical College, Datta Meghe Institute of Higher Education and Research, Wardha, IND

**Keywords:** proton pump inhibitors (ppis), helicobacter pylori infection, peptic ulcer disease (pud), gastroesophageal reflux disease (gerd), gastric acid-related diseases, vonoprazan

## Abstract

Gastric acid-related diseases, including gastroesophageal reflux disease (GERD), peptic ulcer disease (PUD), and *Helicobacter pylori* (*H. pylori*) infection, present significant clinical challenges due to their prevalence and potential for severe complications. Effective management of these conditions is essential for symptom relief, mucosal healing, and prevention of complications. This review aims to evaluate the efficacy and safety of vonoprazan, a novel potassium-competitive acid blocker (P-CAB), in the treatment of gastric acid-related diseases and to compare it with traditional proton pump inhibitors (PPIs). A comprehensive analysis of clinical trials and studies was conducted to assess the effectiveness of vonoprazan in managing GERD, PUD, and *H. pylori* infection. The safety profile of vonoprazan was also reviewed, and comparisons were made to PPIs and other gastric acid suppressants. Vonoprazan demonstrates superior and more consistent acid suppression than PPIs, resulting in rapid and sustained symptom relief and mucosal healing. Clinical trials have shown its efficacy in treating GERD, PUD, and *H. pylori *infection, with higher eradication rates for H. pylori when used in combination therapies. The safety profile of vonoprazan is favorable, with fewer adverse effects and drug interactions compared to PPIs. Vonoprazan offers a promising alternative to traditional PPIs for the management of gastric acid-related diseases. Its unique mechanism of action and superior efficacy make it a valuable option for patients requiring effective and reliable acid suppression. Further research is warranted to explore its potential in broader clinical applications and to establish long-term safety data.

## Introduction and background

Gastric acid-related diseases encompass a variety of conditions that result from the overproduction or improper regulation of gastric acid. These conditions include gastroesophageal reflux disease (GERD), peptic ulcer disease (PUD), and infections with *Helicobacter pylori *(*H. pylori*). GERD is characterized by the backward flow of stomach acid into the esophagus, leading to symptoms such as heartburn, regurgitation, and, in severe cases, esophageal damage [1]. PUD involves the formation of ulcers in the stomach lining or the first part of the small intestine, often causing significant pain and discomfort. *H. pylori *infection is a major cause of chronic gastritis and peptic ulcers, and it can lead to more severe complications like gastric cancer if left untreated [2]. Effective management of gastric acid-related diseases is crucial due to the potential for significant morbidity and, in some cases, mortality associated with these conditions. Chronic GERD can lead to complications such as esophagitis, Barrett's esophagus, and an increased risk of esophageal cancer. Peptic ulcers can result in serious outcomes, including bleeding, perforation, and gastric outlet obstruction [3]. Moreover, untreated *H. pylori *infection can contribute to the development of gastric cancer. Therefore, it is essential to use therapeutic agents that provide symptom relief, promote healing, and prevent complications. The goal of treatment typically involves reducing gastric acid secretion, promoting mucosal healing, and eradicating *H. pylori* infection when present [4].

Vonoprazan is a novel potassium-competitive acid blocker (P-CAB) that has been developed as an alternative to traditional proton pump inhibitors (PPIs) for the management of gastric acid-related diseases [5]. Unlike PPIs, which inhibit the H+/K+ ATPase enzyme in a delayed and often inconsistent manner, vonoprazan directly and competitively blocks the potassium binding site of the enzyme, leading to a more rapid and sustained suppression of gastric acid secretion. This mechanism of action allows vonoprazan to offer more effective and consistent acid suppression, making it a promising option for patients with gastric acid-related conditions [6]. The primary objective of this review is to comprehensively evaluate the efficacy and safety of vonoprazan in managing gastric acid-related diseases. This includes an in-depth analysis of clinical trials and studies that have assessed vonoprazan's effectiveness in treating GERD, PUD, and *H. pylori* infection. Additionally, the review aims to compare the safety profile of vonoprazan with traditional PPIs and other gastric acid suppressants. By synthesizing the available evidence, this review provides clinicians with a detailed understanding of the potential benefits and risks of vonoprazan, thereby aiding in informed decision-making for managing patients with gastric acid-related diseases.

## Review

Pharmacological profile of vonoprazan

Mechanism of Action

**Figure 1:**
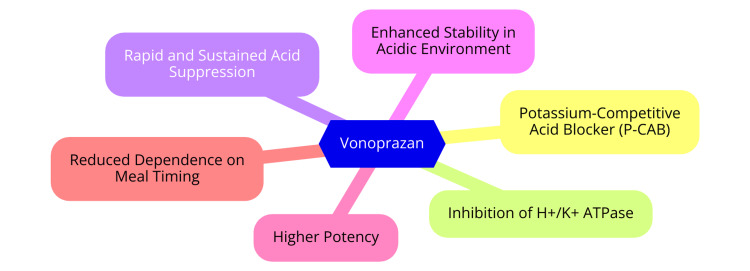
The mechanism of action of vonoprazan Image credit: Dr. Vishal Padwale

Vonoprazan is a P-CAB that inhibits gastric acid secretion by reversibly binding to and blocking the gastric proton pump, known as the H+, K+-ATPase enzyme. Unlike PPIs, vonoprazan does not necessitate acid activation and can deliver rapid and enduring acid suppression [7]. Vonoprazan's mechanism of action differs from PPIs in that it competes with potassium ions to reversibly inhibit H+ and K+-ATPase. In contrast, PPIs exert irreversible action on the proton pump. Vonoprazan's reversible inhibition of H+, K+-ATPase activity results in decreased gastric acid secretion, with 350 times the potency of PPIs [7]. This reversible binding enables vonoprazan to act more swiftly and maintain acid suppression longer than PPIs. Moreover, its ability to inhibit the proton pump across all stages of its catalytic cycle without needing acid activation enhances its potent and sustained acid-suppressing effects [7]. The mechanism of vonoprazan is shown in Figure 1.

Pharmacokinetics

Vonoprazan, a P-CAB, inhibits gastric acid secretion by reversibly binding to and blocking the gastric proton pump, also known as the H+, K+-ATPase enzyme. Unlike PPIs, vonoprazan does not require acid activation and provides rapid and long-lasting acid suppression. Its mechanism of action involves competing with potassium ions to reversibly inhibit H+ and K+-ATPase, whereas PPIs act irreversibly. Vonoprazan's inhibition of H+, K+-ATPase activity results in a gastric acid secretion decrease 350 times more potent than PPIs [7]. After oral administration, vonoprazan is rapidly absorbed, reaching a median time to maximum concentration (Tmax) of 1-2 hours. Its pharmacokinetics show slightly greater than dose-proportional increases in exposure with increasing doses. In rats, vonoprazan exhibits a bioavailability of approximately 9%, suggesting significant first-pass metabolism. With a high volume of distribution exceeding body volume by 10-fold in rats, vonoprazan demonstrates extensive tissue distribution. The drug undergoes primary metabolism by the CYP3A4 and CYP2C19 enzymes [8]. The estimated mean elimination half-life of vonoprazan is up to nine hours. Vonoprazan clearance surpasses hepatic blood flow in rats, indicating the involvement of extrahepatic metabolism. There are no discernible differences in vonoprazan's pharmacokinetics between Japanese and non-Japanese populations. Weight, age, and race are not expected to clinically affect vonoprazan exposure [9].

Comparison with Traditional PPIs

Vonoprazan, a P-CAB, has emerged as a promising alternative to traditional PPIs in treating gastric acid-related disorders, demonstrating superior efficacy in several critical aspects. For first-line *H. pylori* eradication, vonoprazan-based regimens achieve significantly higher eradication rates (89.0% to 97.4%) than PPI-based regimens (69.6% to 82.0%). In treating erosive esophagitis, vonoprazan shows superior healing rates (92.3% to 99.0%) compared to lansoprazole (93.2% to 95.5%). It also outperforms lansoprazole in efficacy for gastric or duodenal ulcers, with eradication rates of 92.6% versus 75.9%. Moreover, vonoprazan proves more effective than PPIs in managing post-endoscopic submucosal dissection (ESD) ulcers, achieving healing rates of 94.9% compared to 78% for PPIs [5]. While vonoprazan demonstrates superior efficacy in multiple areas, a meta-analysis indicates that its efficacy is comparable to PPIs in treating peptic ulcers following ESD, with no statistically significant differences noted. In terms of safety, the short-term profile of vonoprazan appears generally comparable to PPIs, with similar rates of treatment-emergent adverse events (AEs) (33.3% vs. 26.4%). However, concerns exist regarding potential longer-term safety issues due to its potent and sustained acid suppression, which may lead to hypergastrinemia. Caution is particularly advised when using vonoprazan in patients with duodenal ulcers, as safety data in this specific population remains limited [10].

Clinical efficacy of vonoprazan

Treatment of GERD

Vonoprazan, a P-CAB, has shown superior efficacy over PPIs in managing specific gastric acid-related conditions. In first-line *H. pylori* eradication, vonoprazan-based regimens achieve significantly higher eradication rates than PPI-based treatments (89.0%-97.4% vs. 69.6%-82.0%). When treating erosive esophagitis, vonoprazan demonstrates greater efficacy than lansoprazole, with healing rates ranging from 92.3% to 99.0% compared to 93.2% to 95.5% for lansoprazole. For gastric or duodenal ulcers, vonoprazan exhibits superior efficacy to lansoprazole, achieving eradication rates of 92.6% versus 75.9%. In managing post-ESD ulcers, vonoprazan also proves effective, with healing rates of 94.9% compared to 78% for PPIs [11]. The short-term safety profile of vonoprazan is generally comparable to that of PPIs, showing similar rates of treatment-emergent AEs (33.3% vs. 26.4%). However, concerns persist regarding potential longer-term safety issues due to vonoprazan's potent and sustained acid suppression, which can lead to hypergastrinemia. Caution is particularly warranted when using vonoprazan in patients with duodenal ulcers, as safety data in this population remains limited [12]. Vonoprazan demonstrates superior efficacy to PPIs in treating first-line *H. pylori *infection, erosive esophagitis, and gastric/duodenal ulcers while maintaining non-inferiority in other gastric acid-related disorders. While its short-term safety profile aligns with PPIs, further investigation into its long-term safety, especially in specific patient subsets, is necessary [13].

Management of PUD

In treating gastric or duodenal ulcers, vonoprazan has demonstrated superior efficacy over lansoprazole, achieving eradication rates of 92.6% compared to 75.9% for lansoprazole. For post-ESD ulcers, vonoprazan has also shown effectiveness with healing rates of 94.9%, surpassing the 78% rate observed with PPIs [14]. However, findings from a meta-analysis of randomized controlled trials indicate no significant difference in ulcer healing rates at four weeks (RR: 1.09; 95% CI: 0.72-1.65) or eight weeks (RR: 1.01; 95% CI: 0.94-1.08) between vonoprazan and PPIs for ESD-induced ulcers [14]. For patients undergoing long-term NSAID therapy, vonoprazan at doses of 10 mg (3.3%) and 20 mg (3.4%) showed lower proportions of endoscopically confirmed recurrent peptic ulcers within 24 weeks compared to lansoprazole 15 mg (5.5%). The Kaplan-Meier cumulative incidence rates of peptic ulcer recurrence and bleeding were either similar to or lower in the vonoprazan groups compared to the lansoprazole group over 104 weeks [15].

Eradication of H. Pylori Infection

Vonoprazan, a P-CAB, has demonstrated superior efficacy over PPIs in managing specific gastric acid-related conditions. In first-line *H. pylori* eradication, vonoprazan-based regimens achieve significantly higher eradication rates than PPI-based treatments (89.0%-97.4% vs. 69.6%-82.0%) [16]. When treating erosive esophagitis, vonoprazan shows higher efficacy than lansoprazole, with healing rates ranging from 92.3% to 99.0% compared to 93.2% to 95.5% for lansoprazole. For gastric or duodenal ulcers, vonoprazan exhibits superior efficacy to lansoprazole, achieving eradication rates of 92.6% versus 75.9%. Additionally, vonoprazan has proven effective in managing post-ESD ulcers, with healing rates of 94.9% compared to 78% for PPIs [17]. The short-term safety profile of vonoprazan is generally comparable to that of PPIs, showing similar rates of treatment-emergent AEs (33.3% vs. 26.4%). However, there are concerns regarding potential longer-term safety issues due to vonoprazan's potent and sustained acid suppression, which can lead to hypergastrinemia. Caution is particularly advised when using vonoprazan in patients with duodenal ulcers, as safety data in this specific population is limited [13].

Other Gastric Acid-Related Conditions

In addition to its use in treating GERD, vonoprazan, a P-CAB, has demonstrated superior efficacy over PPIs in managing various other gastric acid-related conditions. For *H. pylori* eradication, vonoprazan-based regimens achieve significantly higher success rates compared to PPI-based treatments, ranging from 89.0% to 97.4% versus 69.6% to 82.0%. This underscores vonoprazan's effectiveness in treating this prevalent bacterial infection [18]. Vonoprazan also shows superior efficacy to lansoprazole in treating gastric or duodenal ulcers, with eradication rates of 92.6% compared to 75.9% for lansoprazole, demonstrating its ability to effectively heal these ulcer types [19]. Moreover, vonoprazan has proven effective in managing post-ESD ulcers, with healing rates of 94.9% compared to 78% for PPIs, suggesting it may be a valuable option for treating these challenging ulcers following certain endoscopic procedures [10]. However, there are safety concerns associated with vonoprazan. Long-term use may lead to hypergastrinemia due to its potent and sustained acid suppression. Additionally, caution is recommended when using vonoprazan in patients with duodenal ulcers, as safety data specific to this population is limited [5].

The safety profile of vonoprazan

Short-Term Safety Data

The short-term safety profile of vonoprazan, a P-CAB, appears generally comparable to PPIs. Pooled data indicate that the incidences of any AEs, drug-related AEs, serious AEs, and AEs leading to drug discontinuation with vonoprazan were 20%, 7%, 1%, and 1%, respectively, showing no significant difference compared to patients taking PPIs [11]. Common AEs associated with vonoprazan include hepatic and skin disorders similar to those observed with PPIs. However, vonoprazan is strongly associated with a higher risk of hemorrhagic enterocolitis, with a reporting odds ratio (ROR) of 86.5. Unlike some PPIs, vonoprazan does not show a significant association with interstitial lung disease [20]. Among PPIs, lansoprazole is noted for the highest risk of microscopic colitis, with an ROR of 405. There are some longer-term safety concerns with vonoprazan due to its potent and sustained acid suppression, which can lead to hypergastrinemia. Caution is particularly advised when using vonoprazan in patients with duodenal ulcers, as long-term safety data in this population is limited [20].

Long-Term Safety Data

The long-term safety data for vonoprazan, a P-CAB, suggests a generally favorable profile based on several studies. One study focused on patients receiving long-term nonsteroidal anti-inflammatory drugs (NSAIDs), where more than 85% were able to continue vonoprazan treatment for at least six months, with a low incidence of adverse drug reactions (ADRs) at 0.71%. The study also reported a low incidence of hemorrhagic lesions (1.04%), confirming the safety and effectiveness of vonoprazan in real-world clinical settings [21]. The VISION trial, assessing vonoprazan as a maintenance treatment for healed erosive esophagitis over three years, found no new safety concerns. Histopathological evaluation of the gastric mucosa showed more common parietal, foveolar, and G cell hyperplasia with vonoprazan compared to lansoprazole, but without marked increases over time. No neoplastic changes were identified in either treatment group, providing reassurance on the long-term safety of vonoprazan [22]. A meta-analysis examining vonoprazan safety reported pooled incidences of AEs, drug-related AEs, serious AEs, and AEs leading to drug discontinuation at 20%, 7%, 1%, and 1%, respectively. Most AEs were mild, and the safety profile was comparable to PPIs. Subgroup analyses indicated higher incidences of AEs among patients with PUD compared to those with GERD, *H. pylori* infection, and artificial ulcers after gastric ESD [23]. Another study using data from the FDA adverse event reporting system (FAERS) analyzed long-term safety and identified potential adverse reactions such as increased plateletcrit, benign duodenal neoplasm, gallbladder volvulus, myopathy endocrine issues, and pernio-like erythema. However, these signals were not confirmed as significant concerns. The study emphasized that while vonoprazan may offer advantages, its long-term safety and adverse effects require further investigation [24].

Common Adverse Effects

Common adverse effects associated with vonoprazan encompass a range of gastrointestinal and other issues. Gastrointestinal adverse effects include diarrhea, abdominal pain, nausea, vomiting, flatulence, indigestion (dyspepsia), and GERD. Compared to PPIs, vonoprazan carries a higher risk of hemorrhagic enterocolitis. Additionally, hepatic disorders, such as elevated liver function tests, and skin disorders, including rash, dermatitis, and urticaria, are commonly reported. Other frequent adverse effects include headache, dizziness, fatigue, fever, decreased appetite, and bone fractures. Infections, particularly upper respiratory and urinary tract infections, are also prevalent among vonoprazan users [25]. While the short-term safety profile of vonoprazan appears generally comparable to that of PPIs, concerns arise regarding potential longer-term safety issues due to its potent and sustained acid suppression. Prolonged acid suppression with vonoprazan can lead to hypergastrinemia, which may increase the risk of certain adverse effects over time. Clinicians should be vigilant about these potential risks and monitor patients closely, especially those with pre-existing conditions or who are taking other medications that may interact with vonoprazan [26].

Comparison with the Safety Profiles of PPIs

The safety profile of vonoprazan, a P-CAB, appears generally comparable to that of PPIs in the short term. Both medications share common adverse effects such as hepatic and skin disorders, diarrhea, abdominal pain, nausea, and vomiting. However, vonoprazan is notably associated with a higher risk of hemorrhagic enterocolitis, with an ROR of 86.5, which is significantly elevated compared to PPIs [11]. While the short-term safety profile of vonoprazan appears similar to PPIs, concerns arise regarding potential longer-term safety issues. Vonoprazan's potent and sustained acid suppression can lead to hypergastrinemia, raising potential risks over extended use. The VISION trial, which conducted a three-year interim analysis, found no new safety concerns but did observe an increase in gastric cellular hyperplasia with vonoprazan, necessitating ongoing monitoring [13]. In comparison to specific PPIs, notable differences emerge. For instance, lansoprazole among the PPIs has the highest risk of microscopic colitis, with an ROR of 405. Moreover, while PPIs as a class have been associated with interstitial lung disease, this association has not been observed with vonoprazan [12]. These distinctions underscore the importance of considering the specific safety profiles of acid-suppressive medications when selecting treatment options for patients.

Patient populations and special considerations

Use in Different Age Groups

Vonoprazan has been assessed across different age groups to evaluate its efficacy and safety in various clinical contexts. In studies comparing vonoprazan to PPIs for primary eradication therapy, the success rate was notably higher in the vonoprazan group for patients under 50 years old, achieving 92.6% compared to 67.8% for PPIs. Among patients over 50 years old, vonoprazan also showed higher success rates, albeit less pronounced, with 90.2% success compared to 74.3% for PPIs [27]. However, when examining vonoprazan-based triple therapy in patients over 60, the superiority observed in younger patients did not translate clearly, suggesting potential efficacy differences in the elderly population [28]. The safety profile of vonoprazan was generally comparable to PPIs, with similar rates of treatment-emergent AEs observed across age groups. Specific adverse effects unique to vonoprazan were not identified, and its efficacy remained consistent across the age spectrum compared to PPIs. Furthermore, ongoing research includes a Phase 1 clinical trial recruiting children aged six to 11 years to evaluate the pharmacokinetics, pharmacodynamics, and safety of vonoprazan in treating GERD. This study aims to provide crucial insights into the safety and efficacy of vonoprazan in a younger age group that has not been extensively studied previously [29].

Considerations for Patients with Comorbid Conditions

Vonoprazan, a P-CAB, has shown superiority over PPIs in managing various gastric acid-related diseases while maintaining non-inferiority in others. For instance, vonoprazan-based regimens achieve significantly higher eradication rates for first-line *H. pylori* eradication (89.0%-97.4% vs. 69.6%-82.0%) compared to PPI-based regimens. It also outperforms lansoprazole in treating erosive esophagitis (healing rates of 92.3%-99.0% vs. 93.2%-95.5%) and gastric/duodenal ulcers (eradication rates of 92.6% vs. 75.9%). Additionally, vonoprazan effectively manages post-ESD ulcers with healing rates of 94.9% compared to 78% for PPIs [11]. In terms of safety, vonoprazan demonstrates a generally comparable profile to PPIs, with similar rates of treatment-emergent AEs (33.3% vs. 26.4%). However, concerns exist regarding potential longer-term safety issues due to its potent and sustained acid suppression, potentially leading to hypergastrinemia. Special caution is advised when prescribing vonoprazan to patients with duodenal ulcers due to limited safety data in this population [13]. Vonoprazan exhibits consistent pharmacokinetics and efficacy across Asian and non-Asian populations. While it shows superior efficacy to PPIs across various conditions, including erosive esophagitis, gastric/duodenal ulcers, and post-ESD ulcers, careful consideration is necessary in patients with duodenal ulcers due to limited safety data [26]. The drug's efficacy is consistent across age groups, with better outcomes observed in younger patients than PPIs, although its effectiveness may be less clear in elderly patients over 70 years old [30]. When prescribing vonoprazan, especially to patients with comorbid conditions, careful consideration of potential drug interactions, increased risk of AEs, and the need for close monitoring is essential. This is particularly critical in older patients with multiple comorbidities, necessitating careful patient selection and possibly dose adjustments to optimize safety and efficacy [30].

Potential Drug Interactions

Vonoprazan, as a potent P-CAB, interacts with various medications due to its inhibition of several cytochrome P450 enzymes, including CYP3A4, CYP2C9, CYP2D6, and CYP2B6. This suggests that caution should be exercised when co-administering vonoprazan with drugs metabolized by these enzymes, as vonoprazan may alter their pharmacokinetics and efficacy [31]. Additionally, vonoprazan's potent acid-suppressing effects can reduce the absorption and efficacy of drugs that require an acidic environment for proper absorption, such as atazanavir, erlotinib, and certain azole antifungals. It is advisable to monitor patients closely when vonoprazan is used concomitantly with these medications [32]. On the other hand, vonoprazan has been found to have no clinically significant pharmacokinetic interactions with low-dose aspirin or common NSAIDs like loxoprofen, diclofenac, and meloxicam. Importantly, it does not affect the antiplatelet activity of low-dose aspirin, suggesting that these combinations can generally be used without requiring dose adjustments [33].

Practical considerations for clinical use

Dosage and Administration

Vonoprazan is available in two dosage forms: 10 mg and 20 mg film-coated tablets. The recommended dosage for treating erosive esophagitis is 20 mg once daily for eight weeks. A dosage of 10 mg once daily is recommended for up to six months to maintain healing. In the case of *H. pylori* infection, vonoprazan is combined with antibiotics. For dual therapy, the recommended regimen is 20 mg twice daily with amoxicillin 1,000 mg three times daily for 14 days. Triple therapy involves taking 20 mg of vonoprazan twice daily with amoxicillin 1,000 mg three times daily and clarithromycin 500 mg twice daily for 14 days [34]. Vonoprazan can be taken with or without food, and it is important to swallow the tablets whole without chewing or crushing them. If a dose is missed, specific instructions are provided based on the indication and the time since the missed dose. The pharmacokinetics of vonoprazan are characterized by time-independent behavior, with steady-state concentrations typically reached by days 3-4 of dosing. Vonoprazan has an elimination half-life ranging from 6.8 to 7.9 hours, and it is primarily eliminated through the urine (67%) and feces (31%) [35].

Monitoring and Follow-Up

Healthcare providers should monitor patients receiving vonoprazan due to potential safety concerns. Before initiating vonoprazan therapy and periodically thereafter, magnesium levels should be evaluated, particularly in patients anticipated to undergo prolonged treatment, as hypomagnesemia has been associated with PPIs. Additionally, it is crucial to recognize that symptomatic response to vonoprazan therapy does not exclude the presence of gastric malignancy in adults. Patients experiencing suboptimal response or early symptomatic relapse following completion of vonoprazan treatment, especially elderly patients, should undergo further follow-up and diagnostic testing to rule out gastric malignancy [36]. Acute tubulointerstitial nephritis (TIN) has been reported with vonoprazan use, and if suspected, the medication should be discontinued promptly, and the patient should undergo evaluation. Vonoprazan may also increase the risk of *Clostridioides difficile*-associated diarrhea (CDAD), akin to PPIs, and patients with persistent or worsening diarrhea should be assessed for CDAD. Similar to PPIs, vonoprazan may heighten the risk of osteoporosis-related fractures, particularly with prolonged and high-dose usage. Patients at risk should receive management based on established treatment guidelines. Caution is warranted when administering vonoprazan to patients with duodenal ulcers due to limited safety data in this specific population. Furthermore, careful monitoring for endocrine cell hyperplasia and the potential development of endocrine cell tumors is essential during long-term therapy with vonoprazan [37].

Guidelines and Recommendations for Clinicians

Vonoprazan is recommended as the initial treatment for severe reflux esophagitis. At the same time, for mild cases, both vonoprazan and PPIs are suitable initial therapies, with vonoprazan proposed for maintenance treatment. In Japan, maintenance therapy to prevent erosive reflux disease recurrence involves a daily dose of 10 mg of vonoprazan. For managing *H. pylori* infection, vonoprazan-based dual therapy (20 mg twice daily with amoxicillin 1,000 mg three times daily) and triple therapy (20 mg twice daily with amoxicillin 1,000 mg three times daily plus clarithromycin 500 mg twice daily) are recommended [38]. Vonoprazan exhibits time-independent pharmacokinetics, achieving steady-state concentrations by days 3-4 of dosing, with an elimination half-life ranging from 6.8 to 7.9 hours. Safety considerations advise caution when using vonoprazan in patients with duodenal ulcers due to limited safety data in this population. Long-term safety issues like hypergastrinemia and the potential development of endocrine cell tumors should be monitored. Vonoprazan demonstrates superiority over PPIs in first-line *H. pylori* eradication and treating erosive esophagitis but shows non-inferiority in other gastric acid-related diseases. However, further research is needed to establish comparative efficacy and safety with other P-CABs [39]. Guidelines from the American Gastroenterological Association caution against increasing calcium, vitamin B, or other supplements due to potential safety concerns associated with PPIs. Clinicians should monitor patients carefully and consider potential long-term safety issues when prescribing vonoprazan [39].

## Conclusions

In conclusion, vonoprazan represents a significant advancement in managing gastric acid-related diseases due to its unique mechanism of action and superior efficacy in acid suppression compared to traditional PPIs. Clinical trials and studies have demonstrated its effectiveness in treating conditions such as GERD, PUD, and *H. pylori* infection, offering rapid and sustained symptom relief and mucosal healing. Additionally, vonoprazan's safety profile has been found to be comparable to, if not better than, that of conventional PPIs, with fewer drug interactions and consistent therapeutic outcomes. These findings suggest that vonoprazan is a promising alternative for patients who do not respond adequately to or experience adverse effects from PPIs. As research continues to explore its potential in various gastric acid-related conditions, vonoprazan is poised to become an integral part of modern therapeutic strategies, offering hope for improved management and quality of life for patients suffering from these prevalent and often debilitating diseases.
